# A Metabolomics Analysis of Adiposity and Advanced Prostate Cancer Risk in the Health Professionals Follow-Up Study

**DOI:** 10.3390/metabo10030099

**Published:** 2020-03-10

**Authors:** Barbra A. Dickerman, Ericka M. Ebot, Brian C. Healy, Kathryn M. Wilson, A. Heather Eliassen, Alberto Ascherio, Claire H. Pernar, Oana A. Zeleznik, Matthew G. Vander Heiden, Clary B. Clish, Edward Giovannucci, Lorelei A. Mucci

**Affiliations:** 1 Department of Epidemiology, Harvard T.H. Chan School of Public Health, Boston, MA 02115, USA; enoonan@hsph.harvard.edu (E.M.E.); kwilson@hsph.harvard.edu (K.M.W.); nhahe@channing.harvard.edu (A.H.E.); aascheri@hsph.harvard.edu (A.A.); cpernar@mail.harvard.edu (C.H.P.); egiovann@hsph.harvard.edu (E.G.); lmucci@hsph.harvard.edu (L.A.M.); 2 Department of Neurology, Harvard Medical School, Boston, MA 02115, USA; bchealy@mgh.harvard.edu; 3 Partners Multiple Sclerosis Center, Brigham and Women’s Hospital, Boston, MA 02115, USA; 4 Biostatistics Center, Massachusetts General Hospital, Boston, MA 02114, USA; 5 Channing Division of Network Medicine, Department of Medicine, Brigham and Women’s Hospital and Harvard Medical School, Boston, MA 02115, USA; nhotz@channing.harvard.edu; 6 Department of Nutrition, Harvard T.H. Chan School of Public Health, Boston, MA 02115, USA; 7 Koch Institute for Integrative Cancer Research and Department of Biology, Massachusetts Institute of Technology, Cambridge, MA 02139, USA; mvh@mit.edu; 8 Department of Medical Oncology, Dana-Farber Cancer Institute, Boston, MA 02215, USA; 9 Broad Institute of Massachusetts, Institute of Technology and Harvard, Boston, MA 02142, USA; clary@broadinstitute.org

**Keywords:** adiposity, epidemiology, fat mass, metabolomics, obesity, advanced prostate cancer, waist circumference

## Abstract

Obesity is associated with a higher risk of advanced prostate cancer, but men with the same body mass index (BMI) may differ in their underlying metabolic health. Using metabolomics data from nested case-control studies in the Health Professionals Follow-Up Study, we calculated Pearson correlations between 165 circulating metabolites and three adiposity measures (BMI, waist circumference, and derived fat mass from a validated prediction equation) to identify adiposity-associated metabolites. We used Lasso to further select metabolites for prediction models of adiposity measures, which we used to calculate metabolic scores representing metabolic obesity. In an independent set of 212 advanced prostate cancer cases (T3b/T4/N1/M1 or lethal during follow-up) and 212 controls, we used logistic regression to evaluate the associations between adiposity measures and metabolic scores with risk of advanced disease. All adiposity measures were associated with higher blood levels of carnitines (Pearson *r* range, 0.16 to 0.18) and lower levels of glutamine (*r* = −0.19) and glycine (*r,* −0.29 to −0.20), in addition to alterations in various lipids. No adiposity measure or metabolic score was associated with risk of advanced prostate cancer (e.g., odds ratio for a 5 kg/m^2^ increase in BMI 0.96 (95% CI: 0.73, 1.27) and BMI metabolic score 1.18 (95% CI: 0.57, 2.48)). BMI, waist circumference, and derived fat mass were associated with a broad range of metabolic alterations. Neither adiposity nor metabolic scores were associated with risk of advanced prostate cancer.

## 1. Introduction

Obesity is associated with a higher risk of advanced prostate cancer [1]. However, men with the same body mass index (BMI) may differ in their underlying metabolic health and subsequent disease risk [2,3,4,5]. The integration of metabolomics and anthropometric data offers the potential to better identify men at highest risk of prostate cancer, elucidate underlying mechanisms, and inform the development of targeted intervention strategies.

Previous metabolomics-based studies have identified the metabolic alterations associated with BMI and waist circumference [4,6,7,8,9,10], but few have evaluated fat mass [11,12]. Some of these studies further examined how the identified metabolites individually related to later risk of diabetes and breast cancer [6,8]. No study to date has examined whether obesity-related metabolic alterations could, collectively as a metabolic signature of obesity, facilitate the identification of men at risk of advanced prostate cancer by adding information beyond standard measures of obesity, such as BMI.

Here we evaluate associations between metabolites, adiposity, and advanced prostate cancer. First, we identify the plasma metabolites associated with various adiposity measures (BMI, waist circumference, and derived fat mass). Second, we use selected metabolites to evaluate the association between metabolically defined obesity and risk of advanced prostate cancer, among all men and within subgroups defined by self-reported adiposity measures.

## 2. Results

### 2.1. Population Characteristics

Table 1 shows the baseline characteristics of the 660 eligible men. The mean age at blood draw was 65 years, the mean BMI was 26 kg/m^2^, the mean waist circumference was 96 cm, and the mean derived fat mass was 22 kg. Controls in the Parkinson’s disease study were more likely to be former smokers compared with men in the amyotrophic lateral sclerosis (ALS) and prostate cancer studies. Advanced prostate cancer cases were more likely to be current smokers than their matched controls.

The mean age at advanced prostate cancer diagnosis was 72 years (range, 50–92). Among the 212 men with advanced prostate cancer, 53% had T3b/T4/N1/M1 tumors at diagnosis and the remaining 47% were classified as such because of metastasis or fatal disease. Among the 88% of cases for whom the grade was available, 37% had high-grade tumors (Gleason grade ≥ 8). Among the 85% of cases for whom prostate-specific antigen (PSA) level at diagnosis was available, the median PSA was 8.5 ng/mL. The median time between the blood draw and prostate cancer diagnosis was 5.5 years (range, 0.1–16.2).

### 2.2. Metabolites Associated with Adiposity

We identified 43 metabolites associated (false discovery rate (FDR) *p*-value < 0.05 and |r| ≥ 0.15) with BMI, 38 with waist circumference, and 33 with derived fat mass (Table 2). There was considerable overlap in identified metabolites across adiposity measures; 28 metabolites were associated with all three measures (in the same direction) (Figure S3). All adiposity measures were associated with higher levels of carnitines (r, 0.16 to 0.18) and specific diacylglycerol (DAG) and triacylglycerol (TAG) lipid species (r, 0.16 to 0.24). All were associated with lower levels of glycine (r, −0.29 to −0.20), glutamine (r = −0.19), as well as some cholesterol ester (CE) (r, −0.23 to −0.19), lysophosphatidylcholine (LPC) (r, −0.34 to −0.18), and lysophosphatidylethanolamine (LPE) (r, −0.29 to −0.17). Mixed associations were found for phosphatidylcholine (PC) (r, −0.18 to 0.20).

Associations were similar when restricted to men with information on all three adiposity measures (Table S1). When restricted to fasting samples, only BMI and derived fat mass were associated with lower glycine (r, −0.32 to −0.31) and LPE (r, −0.26 to −0.25), BMI was associated with higher valine (r = 0.23), waist circumference was associated with higher ceramides (r, 0.23 to 0.24), all adiposity measures were associated with higher PC (r, 0.25 to 0.28), and neither glutamine nor carnitines were identified as adiposity-associated metabolites; patterns of associations were otherwise similar, with stronger correlations observed for various DAG and TAG lipid species (r, 0.24 to 0.36) (Table S2).

In Lasso models to derive metabolic scores, there were 15 metabolites with a non-zero coefficient in models for BMI, 15 for waist circumference, and 21 for derived fat mass (File S2). Each model retained at least one of the identified amino acids and the top hit for carnitines, LPC, LPE, and PC, in addition to a selection of lipids.

### 2.3. Metabolically Defined Obesity and Advanced Prostate Cancer Risk

Table 3 shows estimated odds ratios for advanced prostate cancer by self-reported or derived adiposity measures and metabolic scores (predicted adiposity given an individual’s metabolites). Estimates for all continuously modeled measures were close to null, though confidence intervals were wide. No trends were observed across increasing quartiles of these measures. Estimates were similar in sensitivity analyses excluding men diagnosed in the first two years after the blood draw (data not shown).

**Figure 1:**
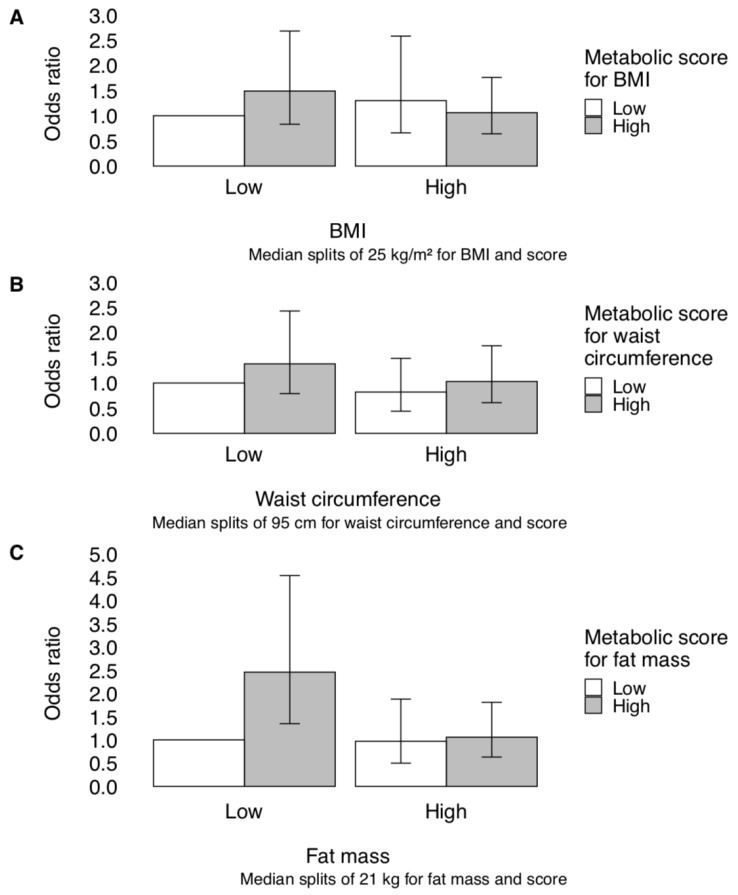
Odds ratios and 95% confidence intervals for advanced prostate cancer by (**a**) BMI-metabolic, (**b**) waist circumference-metabolic, and (**c**) fat mass-metabolic groups. Men were classified into one of four groups based on having a self-reported/derived adiposity measure above versus below the median and a metabolic score (predicted adiposity based on metabolites) above versus below the median. Estimates were adjusted for age, history of diabetes, physical activity, smoking status, family history of prostate cancer, and recent PSA testing.

Figure 1 shows estimated odds ratios for men cross-classified by self-reported or derived adiposity measures and metabolic scores. For waist circumference and derived fat mass, the estimated odds ratio for advanced prostate cancer was highest among men with a low self-reported or derived adiposity measure but a high metabolic score. However, confidence intervals were wide and overlapping.

## 3. Discussion

In this analysis of 165 measured metabolites, we identified a substantial number of metabolites associated with BMI, waist circumference, and derived fat mass, with considerable overlap of metabolites across adiposity measures. In an independent set of men, neither adiposity measures nor metabolic scores representing metabolic obesity were independently associated with the risk of advanced prostate cancer. However, we found suggestive evidence that men with a low waist circumference or fat mass but high metabolic obesity score were at the highest risk of advanced disease, although the power for these analyses was lower.

Among lipid metabolites, we identified inverse associations for CE, LPC, and LPE, positive associations for DAG and TAG lipid species, and mixed associations for PC with adiposity measures. These findings are largely consistent with a cross-sectional study of 217 metabolites among 2383 Framingham Offspring participants, with the exception of CE and TAG, which showed positive and mixed associations in that study, respectively [4]. That study also found that LPC containing an 18:2 fatty acid was the lipid most strongly associated with BMI and waist circumference [4], in line with our findings. A prospective study identified low LPC 18:2 as a predictor of incident pre-diabetes and diabetes over a seven-year period, independent of BMI [13].

We found that carnitines were positively associated with all adiposity measures. Carnitine, which can be endogenously synthesized or absorbed from dietary sources such as meat, plays an important role in metabolism by transporting long-chain fatty acids across mitochondrial membranes, making them essential for fatty acid β-oxidation [14,15]. Acylcarnitine accumulation may result from fatty acid oxidation defects in obese and insulin resistant individuals [16]. We found that waist circumference and derived fat mass were associated with two acylcarnitines not associated with BMI: C16 (L-palmitoylcarnitine) and C12:1 (trans-2-dodecenoylcarnitine). A recent cross-sectional study reported that higher levels of these two metabolites may help to distinguish overweight individuals with high versus low visceral fat area (≥100 versus <100 cm^2^ at L4) [17].

Among amino acids, we identified inverse associations for glutamine and glycine with all adiposity measures, which is consistent with previous findings [4,6,7,8,9,10]. Several studies have also identified positive associations between branched chain amino acids (valine, leucine, and isoleucine) and aromatic amino acids (tyrosine and phenylalanine) with BMI and/or waist circumference [4,6,7,9]. All but isoleucine were included in our study, but we only identified a positive association between BMI and valine when restricting to fasting samples. This may be due to a different distribution of BMI or modifiers, such as diet and physical activity, in our study population compared with others.

Prospective studies have reported associations between glutamine, glycine, and glutamate with future diabetes risk [6,18], and experimental studies have demonstrated that glutamine supplementation in humans and mice leads to improved glucose tolerance [6,19]. This suggests that some of these metabolites may not only be biomarkers of obesity but also effectors of its later sequelae. While we found no evidence for an association between metabolically defined obesity and advanced prostate cancer in our study, the identified metabolites may prove relevant for other disease outcomes.

Prior meta-analyses reported an advanced prostate cancer relative risk of 1.09 (95% CI: 1.02, 1.16; 13 studies) and a prostate cancer-specific mortality relative risk of 1.15 (95% CI: 1.06, 1.25; 6 studies) per 5 kg/m^2^ increase in BMI [1,20]. These estimates are higher than our findings for advanced/fatal prostate cancer (odds ratio 0.96, 95% CI: 0.73, 1.27), but our power was limited by a small sample. Different estimates across studies may also be related to differences in BMI assessment (i.e., timing, self-reported versus measured), definition of advanced disease, and participant characteristics.

Individuals with the same measured obesity may differ in their underlying metabolic health [2,3,4], which could be relevant for later disease risk. We took a novel approach to address this by calculating metabolic scores to summarize underlying metabolic obesity. After cross-classifying men by self-reported and metabolically defined obesity, we found that men at the highest risk of advanced prostate cancer had a low adiposity measure but a high metabolic score. The determinants of a normal weight, metabolically obese profile are unknown but may be related to genetic factors influencing adipocyte function, body fat distribution, and insulin resistance and/or lifestyle factors such as physical activity and diet [5,21]. Men in this group may have a strong propensity for dysregulated metabolism, given their unfavorable metabolic factors despite normal weight, which may contribute to the development and progression of advanced prostate cancer [22]. Further investigation of this phenotype may provide additional insight into the underlying mechanisms and potential intervention targets for clinically important prostate cancer.

The major strength of our study is the integration of metabolomics with detailed clinical and lifestyle data within a well-characterized prospective cohort. This allowed us to investigate several adiposity measures and many metabolites while adjusting for important covariates. It also allowed us to assess adiposity and metabolites before advanced prostate cancer diagnosis to establish a temporal relationship and eliminate the potential for recall bias.

Our study also has some limitations. We relied on self-reported adiposity measures, which are subject to measurement error. However, a previous validation study in the Health Professionals Follow-up Study (HPFS) showed that self-reported weights and waist circumferences were highly correlated with technician-measured values [23]. We also relied on a single measurement of adiposity and metabolites in midlife, and it is possible that long-term average measures are the most biologically relevant for prostate cancer risk. Nonetheless, a pilot study showed that approximately 90% of metabolites were reproducible over two years within individuals [24], and we estimated Pearson correlation coefficients > 0.90 for BMI reports up to six years apart among men in the prostate cancer nested case-control study (data not shown). This suggests that a single measure may be reasonably representative of average values over midlife. Any error in assessing these average values is expected to be independent from the rate of prostate cancer and, therefore, attenuate our estimates. Our power for the analyses of advanced prostate cancer was limited by the sample size. Lastly, our study population consisted of middle-aged or older health professionals of predominantly European ancestry, so our estimates may not be generalizable to other populations with different distributions of adiposity or risk factors.

In summary, we found that BMI, waist circumference, and derived fat mass were associated with a broad range of metabolic alterations, involving lipids, amino acids, and amino acid derivatives. Neither adiposity nor metabolic scores were associated with risk of advanced prostate cancer in this population of men. However, there was suggestive evidence that a subgroup of men with higher measures of metabolic obesity underlying lower measures of waist circumference and fat mass are at a higher risk. The obesity-identified metabolites may inform future integrative-metabolomics research to better identify individuals at the highest risk of disease.

## 4. Materials and Methods

### 4.1. Study Population

The HPFS is an ongoing prospective cohort study of 51,529 US male health professionals aged 40–75 years at enrollment in 1986. The participants reported detailed clinical and lifestyle information at enrollment and every two years thereafter. Blood samples were collected from 18,225 (35%) participants from 1993–1995. The samples were mailed to our laboratory overnight on cold packs and then centrifuged to collect and store plasma in liquid nitrogen freezers. Participants reported the timing of blood collection and fasting status on a questionnaire returned with the samples.

For the current study, we included participants who had provided a blood sample and been previously selected for nested metabolomic case-control analyses in the HPFS. For the identification of adiposity-associated metabolites, we included 236 controls from case-control analyses of amyotrophic lateral sclerosis (ALS) and Parkinson’s disease who had measures of the same metabolites (see also File S1). We selected controls only to minimize the possibility that latent ALS or Parkinson’s disease influenced the metabolite or adiposity measures.

To evaluate metabolically defined obesity and advanced prostate cancer risk, we included an independent set of 212 advanced prostate cancer cases (stage T3b/T4/N1/M1 at diagnosis or development of metastasis or death due to prostate cancer during follow-up) and 212 matched controls. The cases were all men diagnosed with advanced prostate cancer between the time of the blood draw and September 2010. For each case, one control was selected who was alive and cancer-free at the time of the case’s diagnosis. The matching criteria were age (±1 year), recent PSA testing prior to the blood draw (since January 1, 1992; yes/no), and the time of day, season, and year of blood collection.

Prostate cancer diagnoses were self-reported on biennial questionnaires and verified in a standardized review of medical records and pathology reports. We obtained information on subsequent metastasis from prostate-cancer-specific biennial questionnaires sent to all prostate cancer survivors and their physicians. Prostate-cancer-specific deaths were verified through review of medical records and death certificates.

### 4.2. Adiposity Measures and Covariates

We assessed BMI, waist circumference, and derived fat mass in the questionnaire preceding blood collection. Participants reported their weight and height in 1986 and updated their weight every two years thereafter. They reported their waist circumference in 1987 and 1996. A previous validation study in HPFS showed that self-reported weights and waist circumferences were highly correlated with technician-measured values (Pearson *r* = 0.97 and *r* = 0.95, respectively) [23]. We derived fat mass using a prediction equation developed in the National Health and Nutrition Examination Survey (NHANES) [25]. Previous validation in NHANES showed that this equation is highly predictive of dual-energy x-ray absorptiometry (DXA)-measured fat mass (R^2^ = 0.90) [25]. We excluded participants missing adiposity measures for the respective analyses (Figure S1). We also excluded one participant with a BMI > 55 kg/m^2^ from the BMI analyses to prevent this outlier from affecting our analyses. We assessed cigarette smoking, physical activity, and history of diabetes in the questionnaire before blood collection.

### 4.3. Metabolite Profiling

Plasma metabolites were profiled at the Broad Institute (Cambridge, MA, USA) using the liquid chromatography tandem mass spectrometry (LC-MS) methods described previously (see also File S1) [24,26]. A total of 165 known metabolites were analyzed in this study, including lipids (6 cholesterol ester (CE), 11 diacylglycerol (DAG), 8 lysophosphatidylcholine (LPC), 6 lysophosphatidylethanolamine (LPE), 19 phosphatidylcholine (PC), 19 phosphatidylethanolamine (PE), 3 sphingomyelin (SM), and 42 triacylglycerol (TAG)), amino acids, and other small molecules (see File S1 and Figure S2 for details on the metabolite selection). The metabolite peak areas were *ln*-transformed to improve normality and then standardized (to mean = 0, SD = 1) within each project to facilitate analyses across projects.

### 4.4. Statistical Analysis

Among the 236 controls in the ALS and Parkinson’s disease studies, we identified metabolites associated with adiposity by calculating partial Pearson correlations between the 165 metabolites and each adiposity measure, adjusting for age and smoking status at the time of the blood draw. We obtained conservative estimates of false discovery rate (FDR) via the Benjamini‒Hochberg procedure [27]. We carried forward metabolites with an FDR *p*-value < 0.05 and |r| ≥ 0.15. Lasso regression models were used to further select metabolites for prediction models of metabolic scores, which represented predicted adiposity (the dependent variable) given men’s levels of adiposity-associated metabolites (the independent variables). This approach allowed us to account for the relative importance of each identified metabolite in the presence of the others and generate a parsimonious model.

In an independent set of 212 advanced prostate cancer cases and 212 matched controls, we applied these models to predict metabolic scores. For example, the BMI metabolic score ranks men by their expected BMI based on their underlying levels of BMI-associated metabolites. These metabolic scores were not intended to be surrogates of obesity, but rather to provide information about metabolic obesity that may be relevant in the pathophysiology of disease. Their estimation was informed by the relationships between adiposity measures and metabolic profiles observed in the reference population, which are shaped by upstream lifestyle and genetic factors. A high metabolic score can be interpreted as having a metabolic profile consistent with an average man of high adiposity in the reference population.

We used logistic regression to estimate odds ratios and 95% confidence intervals for advanced prostate cancer comparing different levels of each self-reported or derived adiposity measure and metabolic score, fit in separate models. We used unconditional logistic regression for statistical efficiency, adjusting for age, diabetes, physical activity, smoking status, family history of prostate cancer, and recent PSA testing. Additionally adjusting all models for fasting status (≥8 versus <8 h) and adjusting fat mass models for height did not influence our estimates (data not shown).

#### 4.4.1. Subgroup Analyses

We estimated the risk for men cross-classified by their self-reported or derived adiposity measures and metabolic scores, each dichotomized at its median.

#### 4.4.2. Sensitivity Analyses

We repeated the analyses to identify adiposity-associated metabolites after restricting to men with (1) complete information on all adiposity measures and (2) fasting blood samples. We repeated the analyses for advanced prostate cancer after excluding men diagnosed in the first two years after the blood draw to minimize the chance that latent disease influenced their metabolite or adiposity measures.

The study protocol was approved by the institutional review boards of the Brigham and Women’s Hospital and Harvard T.H. Chan School of Public Health, and those of participating registries as required. The analyses were conducted using R, version 3.6.0, and SAS, version 9.4 (SAS Institute, Inc. Cary, NC, USA).

## Figures and Tables

**Table 1:** Baseline characteristics of eligible participants from nested-case control studies of various outcomes, Health Professionals Follow-Up Study, 1993–1996 ^a^.

Characteristic, Mean (SD) or %	Amyotrophic Lateral Sclerosis Study	Parkinson’s Disease Study	Prostate Cancer Study	
	Controls (*n* = 52)	Controls (*n* = 184)	Controls (*n* = 212)	Advanced Cases (*n* = 212)
Age (years)	62.7 (8.3)	65.3 (8.0)	65.3 (8.4)	65.4 (8.5)
Body mass index (kg/m^2^)	25.9 (2.5)	25.5 (2.8)	25.8 (3.6)	25.8 (4.1)
Waist circumference (cm)	94.7 (8.1)	95.5 (8.5)	96.5 (10.3)	95.7 (9.8)
Derived fat mass (kg) ^b^	21.9 (4.3)	21.6 (5.2)	22.5 (6.4)	21.7 (5.9)
Total physical activity (MET hours/week)	39.7 (29.3)	34.4 (27.4)	32.5 (25.6)	31.1 (28.9)
Year of blood donation				
1993–1994	87	94	97	97
1995–1996	14	6	3	3
Fasting ≥ 8 h	50	55	62	67
Race/ethnicity, White	94	98	97	99
Smoking status				
Never	50	39	46	43
Former	46	58	50	51
Current	4	3	4	7
History of diabetes mellitus	4	4	6	7
Family history of prostate cancer	--	--	9	9
Recent prostate-specific antigen testing ^c^	--	--	60	62

Abbreviation: MET, Metabolic equivalent of task. Frequencies of polytomous variables may not add up to 100% due to rounding. ^a^ Baseline characteristics were measured at the time of blood draw when possible, and otherwise in the questionnaire preceding blood draw. ^b^ Derived fat mass from validated anthropometric prediction models developed in the National Health and Nutrition Examination Survey (NHANES). ^c^ Prostate-specific antigen testing since January 1, 1992.

**Table 2:** Metabolites associated with adiposity measures (FDR *p*-value < 0.05 and |r| ≥ 0.15) among controls (*n* = 219–234) ^a^ from nested case-control studies, Health Professionals Follow-Up Study, 1993–1996.

HMDB ID ^b^	Metabolite Name	Body Mass Index		Waist Circumference		Derived Fat Mass	
		Pearson Correlation Coefficient ^c^	FDR *p*-value	Pearson Correlation Coefficient ^c^	FDR *p*-value	Pearson Correlation Coefficient ^c^	FDR *p*-value
**Amino acids**							
HMDB00123	glycine	**−0.29**	**<0.001**	−0.20	0.02	**−0.25**	**0.01**
HMDB00641	glutamine	**−0.19**	**0.02**	**−0.19**	**0.03**	**−0.19**	**0.04**
**Carnitines**							
HMDB06347	C26 carnitine	**0.18**	**0.03**	**0.18**	**0.04**	--	--
HMDB00688	C5 carnitine	0.18	0.03	--	--	--	--
HMDB00705	C6 carnitine	0.18	0.03	**0.18**	**0.03**	--	--
HMDB02013	C4 carnitine	**0.16**	**0.05**	--	--	--	--
HMDB13326	C12:1 carnitine	--	--	**0.17**	**0.04**	--	--
HMDB00222	C16 carnitine	--	--	--	--	**0.18**	**0.04**
**Lipids**							
***CE***							
HMDB10375	C22:5 CE	−0.20	0.01	**−0.22**	**0.01**	**−0.23**	**0.01**
HMDB06733	C22:6 CE	--	--	--	--	**−0.19**	**0.03**
***DAG***							
*Saturated*							
HMDB07098	C32:0 DAG	**0.23**	**0.01**	0.21	0.01	**0.22**	**0.01**
*Unsaturated*							
HMDB07102	C34:1 DAG	0.24	<0.01	**0.24**	**0.01**	**0.24**	**0.01**
HMDB07099	C32:1 DAG	0.23	0.01	**0.24**	**0.01**	0.24	0.01
HMDB07103	C34:2 DAG	0.22	0.01	0.23	0.01	**0.23**	**0.01**
HMDB07132	C34:3 DAG	0.22	0.01	0.23	0.01	**0.22**	**0.01**
HMDB07218	C36:2 DAG	0.21	0.01	0.23	0.01	0.22	0.01
HMDB07216	C36:1 DAG	0.21	0.01	**0.22**	**0.01**	0.23	0.01
HMDB07219	C36:3 DAG	0.20	0.02	0.21	0.01	**0.20**	**0.02**
HMDB07248	C36:4 DAG	**0.17**	**0.04**	0.17	0.04	--	--
HMDB07199	C38:5 DAG	**0.16**	**0.05**	--	--	--	--
***LPC***							
HMDB10386	C18:2 LPC	**−0.34**	**<0.0001**	**−0.23**	**0.01**	−0.29	<0.001
HMDB10397	C20:5 LPC	**−0.34**	**<0.0001**	−0.24	0.01	**−0.29**	**<0.001**
HMDB02815	C18:1 LPC	−0.29	<0.001	**−0.21**	**0.01**	**−0.26**	**0.01**
HMDB10404	C22:6 LPC	−0.25	<0.01	**−0.18**	**0.03**	**−0.26**	**0.01**
***LPE***							
HMDB11503	C16:0 LPE	**−0.29**	**<0.001**	**−0.17**	**0.04**	**−0.23**	**0.01**
HMDB11507	C18:2 LPE	**−0.26**	**<0.01**	--	--	--	--
HMDB11506	C18:1 LPE	−0.22	0.01	--	--	−0.18	0.04
HMDB11130	C18:0 LPE	−0.20	0.02	--	--	--	--
HMDB11526	C22:6 LPE	−0.19	0.02	--	--	--	--
***PC***							
HMDB11210	C34:2 PC plasmalogen	−0.16	0.05	--	--	--	--
HMDB08047	C38:3 PC	**0.20**	**0.01**	**0.20**	**0.02**	--	--
HMDB08057	C40:6 PC	0.19	0.02	0.19	0.03	--	--
HMDB08511	C40:10 PC	**−0.16**	**0.05**	--	--	**−0.18**	**0.04**
***TAG***							
*Unsaturated*							
HMDB05369	C52:2 TAG	0.23	0.01	0.23	0.01	0.23	0.01
HMDB05360	C50:1 TAG	0.23	0.01	0.21	0.01	0.22	0.01
HMDB05384	C52:3 TAG	0.22	0.01	0.22	0.01	**0.21**	**0.02**
HMDB05433	C50:3 TAG	0.22	0.01	0.23	0.01	0.22	0.01
HMDB05377	C50:2 TAG	0.22	0.01	0.21	0.01	0.22	0.01
HMDB05367	C52:1 TAG	**0.20**	**0.01**	0.20	0.02	**0.21**	**0.01**
HMDB05376	C48:2 TAG	0.19	0.02	0.20	0.02	0.21	0.02
HMDB05432	C48:3 TAG	0.18	0.03	0.20	0.02	**0.20**	**0.02**
HMDB10412	C46:1 TAG	**0.18**	**0.03**	0.17	0.04	**0.20**	**0.02**
HMDB05403	C54:2 TAG	0.18	0.03	**0.20**	**0.02**	0.20	0.02
HMDB05363	C52:4 TAG	0.18	0.03	0.18	0.03	--	--
HMDB05359	C48:1 TAG	0.17	0.04	0.17	0.04	**0.18**	**0.04**
HMDB10419	C46:2 TAG	0.17	0.05	0.17	0.04	0.19	0.04
HMDB05435	C50:4 TAG	--	--	0.17	0.04	--	--
HMDB05405	C54:3 TAG	--	--	0.18	0.04	--	--
**Purine nucleosides**							
HMDB03331	1-methyladenosine	--	--	**0.18**	**0.03**	**0.18**	**0.04**

Abbreviations: CE, cholesterol ester; DAG, diacylglycerol; LPC, lysophosphatidylcholine; LPE, lysophosphatidylethanolamine; PC, phosphatidylcholine; TAG, triacylglycerol. Boldface indicates a metabolite with a non-zero coefficient when all metabolites associated with that adiposity measure were entered into Lasso. ^a^ The number of men contributing to each analysis was 234 for BMI, 233 for waist circumference, 219 for derived fat mass. ^b^ Representative HMDB IDs provided for PC, DAG, and TAG lipids. ^c^ Estimates from partial Pearson correlation, adjusted for age (continuous) and smoking status (ever/never).

**Table 3:** Estimated odds ratios ^a^ for advanced prostate cancers by self-reported or derived adiposity and metabolic scores (predicted adiposity given an individual’s metabolites), Health Professionals Follow-Up Study.

Adiposity Measure	Self-reported or Derived			Metabolic Score		
	Cases/ Total	Odds Ratio	95% CI	Cases/ Total	Odds Ratio	95% CI
**Body mass index (kg/m^2^) ^b^**						
per 5 kg/m^2^ increase	201/409	0.96	(0.73, 1.27)	201/409	1.18	(0.57, 2.48)
Quartile 1	46/104	1.00	--	49/103	1.00	--
Quartile 2	60/102	1.78	(1.02, 3.13)	40/102	0.71	(0.40, 1.23)
Quartile 3	49/102	1.14	(0.65, 1.98)	60/102	1.57	(0.90, 2.75)
Quartile 4	46/101	1.04	(0.60, 1.82)	52/102	1.12	(0.64, 1.97)
*p*-trend			0.72			0.20
**Waist circumference (cm) ^c^**						
per 1 SD increase	200/408	0.90	(0.73, 1.10)	200/408	0.99	(0.81, 1.21)
Quartile 1	53/110	1.00	--	45/102	1.00	--
Quartile 2	52/96	1.25	(0.72, 2.19)	52/102	1.35	(0.77, 2.36)
Quartile 3	52/110	0.93	(0.54, 1.60)	53/102	1.38	(0.79, 2.43)
Quartile 4	43/92	0.90	(0.50, 1.59)	50/102	1.19	(0.67, 2.12)
*p*-trend			0.52			0.54
**Derived fat mass (kg) ^d^**						
per 1 SD increase	193/388	0.89	(0.73, 1.10)	193/388	1.07	(0.82, 1.39)
Quartile 1	49/97	1.00	--	42/97	1.00	--
Quartile 2	54/97	1.20	(0.68, 2.13)	51/97	1.45	(0.82, 2.58)
Quartile 3	44/97	0.78	(0.44, 1.38)	54/97	1.63	(0.92, 2.91)
Quartile 4	46/97	0.84	(0.47, 1.50)	46/97	1.15	(0.64, 2.06)
*p*-trend			0.30			0.54

Abbreviation: CI, confidence interval. ^a^ Estimates from unconditional logistic regression models adjusted for age (years, continuous), history of diabetes (yes/no), physical activity (MET hours/week, continuous), smoking status (ever/never), family history of prostate cancer (yes/no), and recent PSA testing (yes/no). Self-reported/derived adiposity measures and metabolic scores were fit in separate models. ^b^ Quartiles of self-reported BMI: [18.6, 23.2], (23.2, 25.2], (25.2, 27.5], (27.5, 42.4]. The fourth quartile (101 men, of whom 46 had advanced prostate cancer) includes 45 men with a BMI >30 kg/m^2^, of whom 22 had advanced prostate cancer. Quartiles of BMI metabolic score: [20.8, 24.6], (24.6, 25.5], (25.5, 26.3], (26.3, 29.7]. ^c^ Standard deviation of self-reported waist circumference: 10.1 cm. Standard deviation of waist circumference metabolic score: 3.4 cm. Quartiles of self-reported waist circumference: [73.7, 88.9], (88.9, 94.6], (94.6, 102.0], (102.0, 135.0]. Quartiles of waist circumference metabolic score: [82.7, 92.9], (92.9, 95.3], (95.3, 97.2], (97.2, 106.0]. ^d^ Standard deviation of derived fat mass: 6.1 kg. Standard deviation of fat mass metabolic score: 2.9 kg. Quartiles of derived fat mass: [7.9, 17.8], (17.8, 21.3], (21.3, 25.1], (25.1, 45.9]. Quartiles of fat mass metabolic score: [13.7, 19.9], (19.9, 21.7], (21.7, 23.5], (23.5, 30.8].

## Supplementary Materials

The following are available online at https://www.mdpi.com/2218-1989/10/3/99/s1, File S1. Detail on metabolite profiling, excluded metabolites, included metabolites, File S2. Lasso models used for predicting adiposity using associated metabolites, Figure S1. Flowchart of participant selection, Figure S2. Flowchart of metabolite selection, Figure S3. Overlap in identified metabolites across adiposity measures (from Main Table 2), Table S1. Metabolites associated with adiposity measures (FDR *p*-value < 0.05 and |r| ≥ 0.15) among 217 controls from nested case-control studies with data on all three adiposity measures, Health Professionals Follow-Up Study, 1993–1996, Table S2. Metabolites associated with adiposity measures (FDR *p*-value < 0.05 and |r| ≥ 0.15) among controls (*n* = 118–126) ^a^ from nested case-control studies with fasting (≥8 h) blood samples, Health Professionals Follow-Up Study, 1993–1996.
