# Supplementary material for: A Metabolomics Analysis of Adiposity and Advanced Prostate Cancer Risk in the Health Professionals Follow-Up Study
Source: Metabolites. 2020 Mar 10;10(3):99. doi: 10.3390/metabo10030099 (PMC7142752; doi:10.3390/metabo10030099)
Supplement: Supplementary file 1 [file metabolites-10-00099-s001.pdf]

Supplementary Materials

File S1

Detail on metabolite profiling …… 2

Detail on excluded metabolites …… 4

Detail on included metabolites …… 8

Figure S1 Flowchart of participant selection …… 10

Figure S2 Flowchart of metabolite selection …… 11

Figure S3 Overlap in identified metabolites across adiposity measures …… 14

Table S1 Metabolites associated with adiposity measures, restricting to men with data on all three adiposity measures …… 15

Table S2 Metabolites associated with adiposity measures, restricting to men with fasting blood samples …… 18

File S2. Lasso models used for predicting adiposity using associated metabolites …… 21

File S1.

Detail on metabolite profiling

Plasma metabolites were profiled at the Broad Institute (Cambridge, MA) using liquid chromatography tandem mass spectrometry (LC-MS) methods previously described.[15,16] Briefly, nontargeted, positive ion mode analyses of polar metabolites were conducted using a Nexera X2 U-HPLC system (Shimadzu Scientific Instruments; Marlborough, MA) coupled to a Q Exactive orbitrap mass spectrometer (Thermo Fisher Scientific; Waltham, MA). Polar metabolites were extracted plasma (10 µL) using 90µL of 74.9:24.9:0.2 v/v/v acetonitrile/methanol/formic acid containing stable isotope-labeled internal standards (valine-d8, Isotec; and phenylalanine-d8, Cambridge Isotope Laboratories; Andover, MA). The extracts were centrifuged (10 min, 9,000 x g, 4°C), and the supernatants were injected onto a 150 x 2 mm Atlantis HILIC column (Waters; Milford, MA). The column was eluted isocratically at a flow rate of 250 µL/min with 5% mobile phase A (10 mM ammonium formate and 0.1% formic acid in water) for 1 minute followed by a linear gradient to 40% mobile phase B (acetonitrile with 0.1% formic acid) over 10 minutes. Polar metabolite MS analyses were carried out using electrospray ionization in the positive ion mode using full scan analysis over m/z 70-800 at 70,000 resolution and 3 Hz data acquisition rate. Additional MS settings were: ion spray voltage, 3.5 kV; capillary temperature, 350°C; probe heater temperature, 300 °C; sheath gas, 40; auxiliary gas, 15; and S-lens RF level 40. Polar metabolite identities were confirmed using authentic reference standards. Lipids were profiled using an LC-MS system comprised of a Shimadzu Nexera X2 U-HPLC (Shimadzu Corp.; Marlborough, MA) coupled to an Exactive Plus orbitrap mass spectrometer (Thermo Fisher Scientific; Waltham, MA). Plasma samples (10 µL) were extracted for lipid analyses using 190 µL of isopropanol containing 1,2-didodecanoyl-sn-glycero-3-phosphocholine as quality control internal standard (Avanti Polar Lipids; Alabaster, AL). After centrifugation, supernatants were injected directly onto a 100 x 2.1 mm, 1.7 µm ACQUITY BEH C8 column (Waters; Milford, MA). The column was eluted isocratically with 80% mobile phase A (95:5:0.1 vol/vol/vol 10mM ammonium acetate/methanol/formic acid) for 1 minute followed by a linear gradient to 80% mobile-phase B (99.9:0.1 vol/vol methanol/formic acid) over 2 minutes, a linear gradient to 100% mobile phase B over 7 minutes, then 3 minutes at 100% mobile-phase B. MS analyses were carried out using electrospray ionization in the positive ion mode using full scan analysis over 200–1000 m/z at 70,000 resolution and 3 Hz data acquisition rate. Other MS settings were: sheath gas 50, in source CID 5 eV, sweep gas 5, spray voltage 3 kV, capillary temperature 300°C, S-lens RF 60, heater temperature 300°C, microscans 1, automatic gain control target 1e6, and maximum ion time 100 ms. Lipid identities were determined based on comparison to reference plasma extracts and were denoted by total number of carbons in the lipid acyl chain(s) and total number of double bonds in the lipid acyl chain(s). Raw data were processed using Progenesis QI software (NonLinear Dynamics) for feature alignment, nontargeted signal detection, and signal integration. Targeted processing of a subset of known metabolites was conducted using TraceFinder software (Thermo Fisher Scientific; Waltham, MA). Metabolite levels were reported as measured LC-MS peak areas, which are proportional to metabolite concentration.

A pilot study in the HPFS and Nurses' Health Study assessed the reliability of this platform using our archival plasma samples as well as within-person variability.[16] Most metabolites performed well, particularly lipids and lipid metabolites (coefficient of variation [CV] =8%, range 1-14%). Fasting status had little influence on the CVs. Almost 90% of metabolites were stable over two years within individuals.

Among known metabolites, we excluded 89 metabolites with mean CV>25% or ICC<0.4 based on quality control samples embedded in each project, 12 metabolites with undetectable levels for ≥10% of participants, and 42 metabolites that were not reproducible over delays in processing of blood samples (Spearman correlation and intraclass correlation coefficient <0.75, comparing immediate and 24-h delayed processing). We restricted to metabolites measured across all nested case-control studies, matching by HMDBID. This left 165 known metabolites for analysis. **Figure S2** includes details on metabolite selection. For metabolites with undetectable levels for <10% of participants, we imputed half the lowest value of the metabolite if the participant's sample was missing ≤25% of metabolites (which applied in all cases). Metabolite peak areas were *ln*-transformed to improve normality and then standardized (to mean=0, SD=1) within each project to facilitate analyses across projects.

Excluded metabolites before restricting to common metabolites across endpoints

Basis for exclusion	Metabolites
CV>25% or ICC<0.4<br>(n=89)	C36:4 hydroxy-PC-A<br>C32:0 PE<br>1-methylhistamine<br>21-deoxycortisol<br>3-(N-acetyl-L-cystein-S-yl) acetaminophen<br>3-hydroxyanthranilic acid<br>3-methylhistidine<br>4-guanidinobutanoic acid<br>5-acetylamino-6-formylamino-3-methyluracil<br>5-hydroxytryptophan_isomer<br>acetaminophen glucuronide<br>acetyl-galactosamine<br>acetylglycine<br>adenosine<br>allantoin<br>aminoisobutyric acid<br>anserine<br>atenolol<br>beta-alanine<br>C16:1 LPC<br>C16:1 MAG<br>C18:0 MAG<br>C18:1-OH carnitine<br>C18:2 SM<br>C20 carnitine<br>C22:1 MAG<br>C22:4 CE<br>C3-DC-CH3 carnitine<br>C32:0 PE<br>C32:2 DAG<br>C34:0 PI<br>C34:1 DAG<br>C34:2 PC<br>C34:2 PE plasmalogen<br>C34:5 PC plasmalogen<br>C36:0 DAG<br>C36:3 PS plasmalogen<br>C36:4 hydroxy-PC-A<br>C38:3 PE plasmalogen<br>C40:6 PE

	C44:13 PE plasmalogen<br>C47:0 TAG<br>C49:0 TAG<br>C5-DC carnitine<br>C54:10 TAG<br>C54:9 TAG<br>C60:12 TAG<br>C7 carnitine<br>cinnamoylglycine<br>citrulline<br>dehydrophytosphingosine<br>deoxycortisone<br>GABA<br>guanidoacetic acid<br>guanine_isomer<br>histamine<br>homocitrulline<br>homocysteine<br>hydroxyectoine<br>hydroxyproline<br>hypotaurine<br>indoleacetic acid<br>isoleucine<br>kynurenic acid<br>L-threo-sphingosine<br>metformin<br>methionine<br>methionine sulfoxide<br>metoprolol<br>N-acetylaspartic acid<br>N-carbamoyl-beta-alanine<br>NH4_C34:3 DAG<br>NH4_C38:5 DAG<br>NH4_C44:2 TAG<br>NH4_C46:3 TAG<br>pantothenol<br>phosphocholine<br>putrescine<br>pyridoxal<br>S-methyl-L-cysteine-S-oxide<br>SDMA<br>serotonin<br>sphingosine

	sphingosine_isomer1<br>sphingosine_isomer2<br>sulfamethoxazole<br>thiamine<br>urobilinogen<br>urobilinogen_isomer<br>urocanic acid<br>xanthosine
Missing for ≥10% of participants<br>(n=12)	C32:2 DAG<br>3-(N-acetyl-L-cystein-S-yl) acetaminophen<br>5-hydroxytryptophan_isomer<br>acetaminophen glucuronide<br>atenolol<br>C30:0 DAG<br>C40:6 PE<br>cotinine<br>hydroxycotinine<br>metoprolol<br>myristoleic acid<br>quinine
Failed delayed processing pilot<br>(n=42)	alpha-glycerophosphocholine<br>arginine<br>C14:0 CE<br>C16:0 CE<br>C16:0 LPC<br>C16:0 SM<br>C18:0 LPC<br>C18:0 MAG<br>C18:0 SM<br>C18:1 CE<br>C18:2 CE<br>C18:3 CE<br>C20:0 SM<br>C20:4 CE<br>C22:0 SM<br>C22:1 MAG<br>C24:0 SM<br>C24:1 SM<br>C32:0 PC<br>C32:2 DAG<br>C34:0 PC

	C34:1 PC plasmalogen-A<br>C34:2 PC<br>C34:3 PC<br>C34:3 PC plasmalogen<br>C34:4 PC<br>C36:0 DAG<br>C36:0 PC<br>C36:2 PC<br>C36:3 PC<br>C36:3 PC plasmalogen<br>C36:4 PC-A<br>C36:5 PC plasmalogen-B<br>C38:2 PC<br>C38:2 PE<br>C38:4 PC<br>C38:6 PC plasmalogen<br>C40:7 PC plasmalogen<br>glutamate<br>histamine<br>sphingosine<br>thiamine

The 165 included metabolites and their categories after restricting to common metabolites across endpoints

Category	Metabolites
Amino acid	Alanine, asparagine, glutamine, glycine, histidine, leucine, lysine, ornithine, phenylalanine, serine, threonine, tryptophan, tyrosine, valine
Carnitine	C10 carnitine (decanoylcarnitine), C10:2 carnitine (2-trans,4-cis-decadienoylcarnitine), C12 carnitine (dodecanoylcarnitine), C12:1 carnitine (trans-2-dodecenoylcarnitine), C14 carnitine (tetradecanoylcarnitine), C14:1 carnitine (cis-5-tetradecenoylcarnitine), C14:2 carnitine (3, 5-tetradecadiencarnitine), C18 carnitine (stearoylcarnitine), C18:1 carnitine (oleoylcarnitine), C18:2 carnitine (linoleyl carnitine), C2 carnitine (L-acetylcarnitine), C26 carnitine (hexacosanoyl carnitine), C3 carnitine (propionylcarnitine), C4 carnitine (butyrylcarnitine), C4-OH carnitine (3-hydroxybutyrylcarnitine), C5 carnitine (isovalerylcarnitine), C5:1 carnitine (tiglylcarnitine), C6 carnitine (hexanoylcarnitine), C8 carnitine (L-octanoylcarnitine), C9 carnitine (nonanoylcarnitine), C16 carnitine (L-palmitoylcarnitine), Carnitine (L-carnitine)
Cholesteryl ester (CE)	C16:1 CE, C18:0 CE, C20:3 CE, C20:5 CE, C22:5 CE, C22:6 CE
Ceramide	C16:0 Ceramide (d18:1), C22:0 Ceramide (d18:1), C24:0 Ceramide (d18:1), C24:1 Ceramide (d18:1)
Diacylglycerol (DAG)	C32:0 DAG, C32:1 DAG, C34:1 DAG, C34:2 DAG, C34:3 DAG, C36:1 DAG, C36:2 DAG, C36:3 DAG, C36:4 DAG, C38:4 DAG, C38:5 DAG
Lysophosphatidylcholine (LPC)	C14:0 LPC, C16:1 LPC, C18:1 LPC, C18:2 LPC, C20:3 LPC, C20:4 LPC, C20:5 LPC, C22:6 LPC
Lysophosphatidylethanolamine (LPE)	C16:0 LPE, C18:0 LPE, C18:1 LPE, C18:2 LPE, C20:4 LPE, C22:6 LPE
Phosphatidylcholine (PC)	C34:2 PC plasmalogen, C36:1 PC plasmalogen, C36:2 PC plasmalogen, C36:4 PC plasmalogen, C36:5 PC plasmalogen-A, C38:4 PC plasmalogen, C38:7 PC plasmalogen, C30:0 PC, C30:1 PC, C32:1 PC, C32:2 PC, C34:1 PC, C36:1 PC, C36:4 PC-B, C38:3 PC, C38:6 PC, C40:10 PC, C40:6 PC, C40:9 PC
Phosphatidylethanolamine (PE)	C34:3 PE plasmalogen, C36:2 PE plasmalogen, C36:3 PE plasmalogen, C36:4 PE plasmalogen, C36:5 PE plasmalogen, C38:5 PE plasmalogen, C38:6 PE plasmalogen, C38:7 PE plasmalogen, C40:7 PE plasmalogen, C34:0 PE, C34:2 PE, C36:0 PE, C36:1 PE, C36:2 PE, C36:3 PE, C36:4 PE, C38:4 PE, C38:5 PE, C38:6 PE
Sphingomyelin (SM)	C14:0 SM, C18:2 SM, C22:1 SM

Triacylglycerol (TAG)		C44:0 TAG, C46:0 TAG, C48:0 TAG, C50:0 TAG, C52:0 TAG, C46:1 TAG, C46:2 TAG, C48:1 TAG, C48:2 TAG, C48:3 TAG, C50:1 TAG, C50:2 TAG, C50:3 TAG, C50:4 TAG, C50:5 TAG, C50:6 TAG, C52:1 TAG, C52:2 TAG, C52:3 TAG, C52:4 TAG, C52:6 TAG, C52:7 TAG, C54:1 TAG, C54:2 TAG, C54:3 TAG, C54:4 TAG, C54:5 TAG, C54:6 TAG, C54:7 TAG, C54:8 TAG, C56:10 TAG, C56:2 TAG, C56:3 TAG, C56:5 TAG, C56:6 TAG, C56:7 TAG, C56:8 TAG, C56:9 TAG, C58:11 TAG, C58:7 TAG, C58:8 TAG, C58:9 TAG
Others	Nitrogenous organic acid	creatine
	Amino acid glycine derivative	dimethylglycine
	Organic compound, constituent of bile	taurine
	Metabolite of nicotinamide	1-methylnicotinamide
	Carboxylic acid of piperidine	pipecolic acid
	TMAO	trimethylamine-N-oxide
	Purine nucleoside	1-methyladenosine
	Phosphatidylserine	C34:0 PS, C40:6 PS
	N-monomethyl-L-arginine	NMMA
	NG,NG-Dimethyl-L-arginine	ADMA

Figure S1 Flowchart of participant selection

BMI analysis

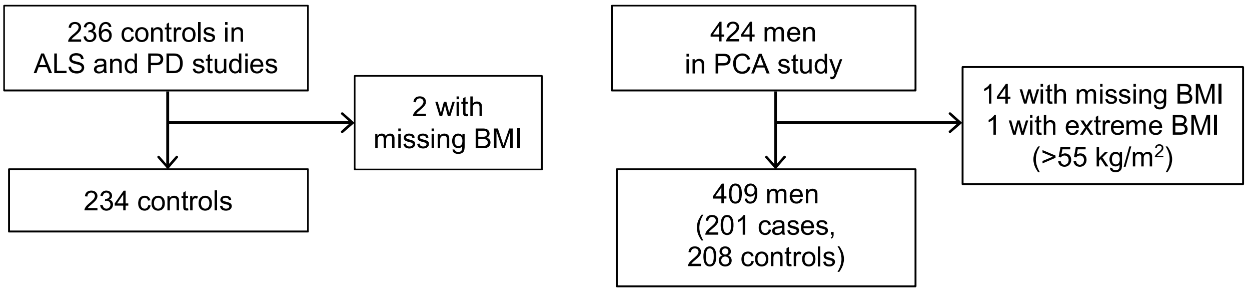

Waist circumference analysis

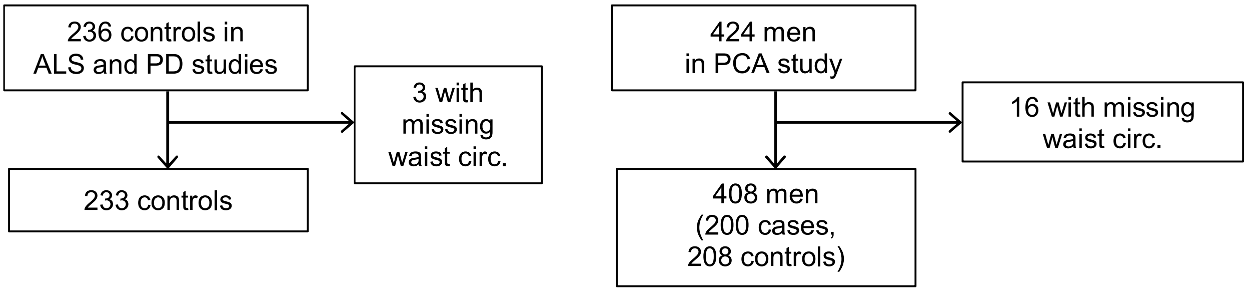

Derived fat mass analysis

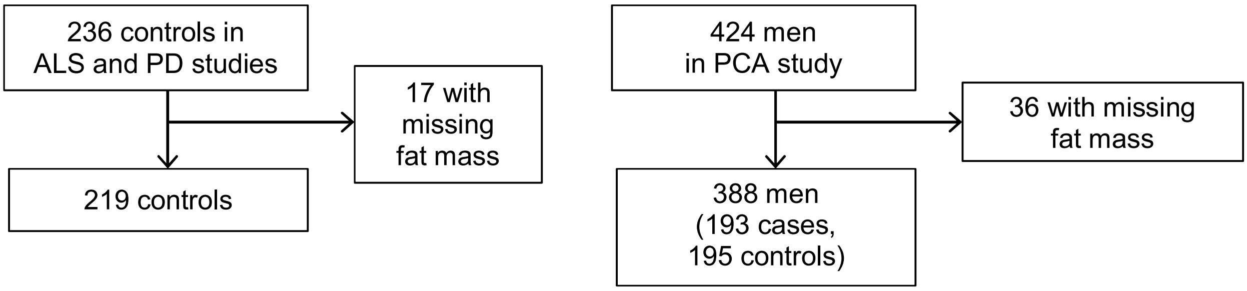

Figure S2 Flowchart of metabolite selection

ALS nested case-control study

C8-positive method (polar and non-polar lipids)

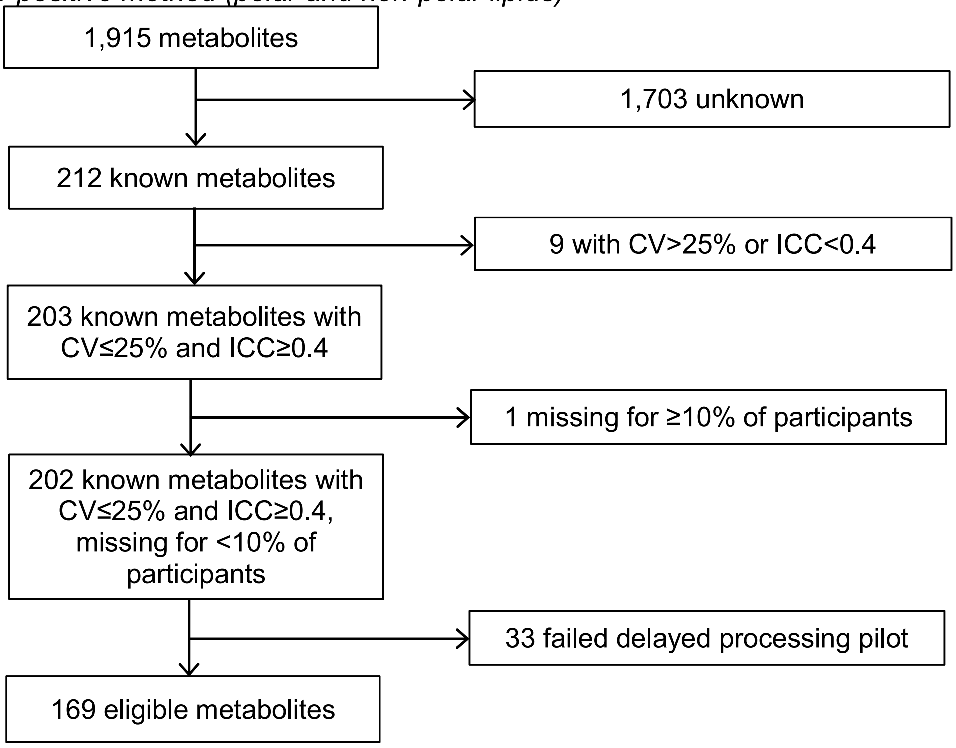

HILIC-positive method (amino acids, dipeptides, and other cationic metabolites)

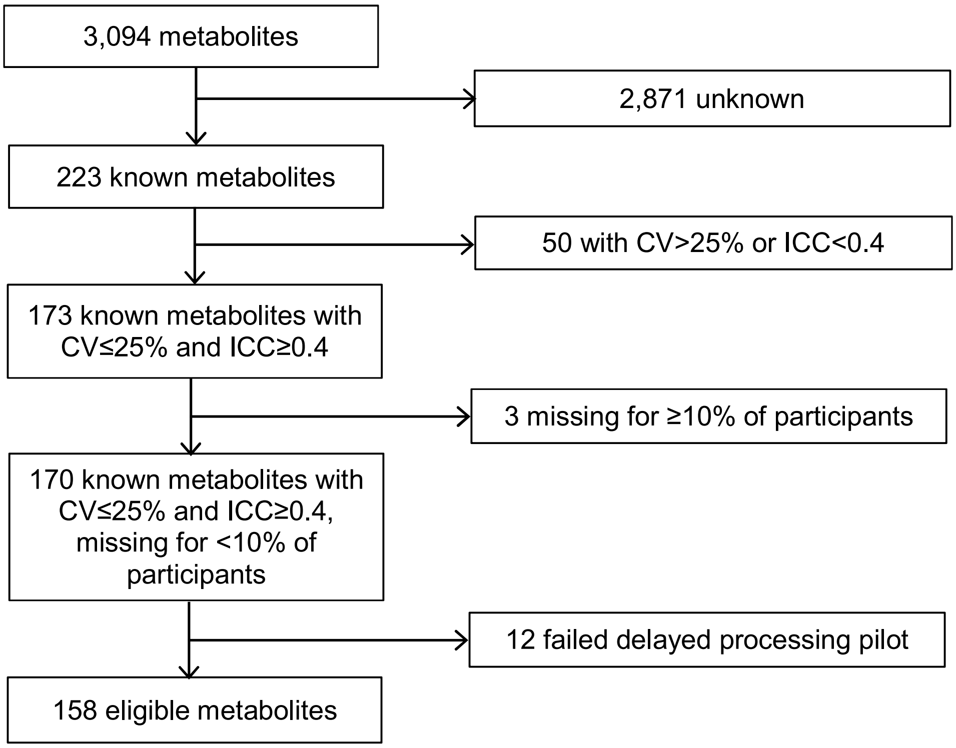

PD nested case-control study

C8-positive method (polar and non-polar lipids)

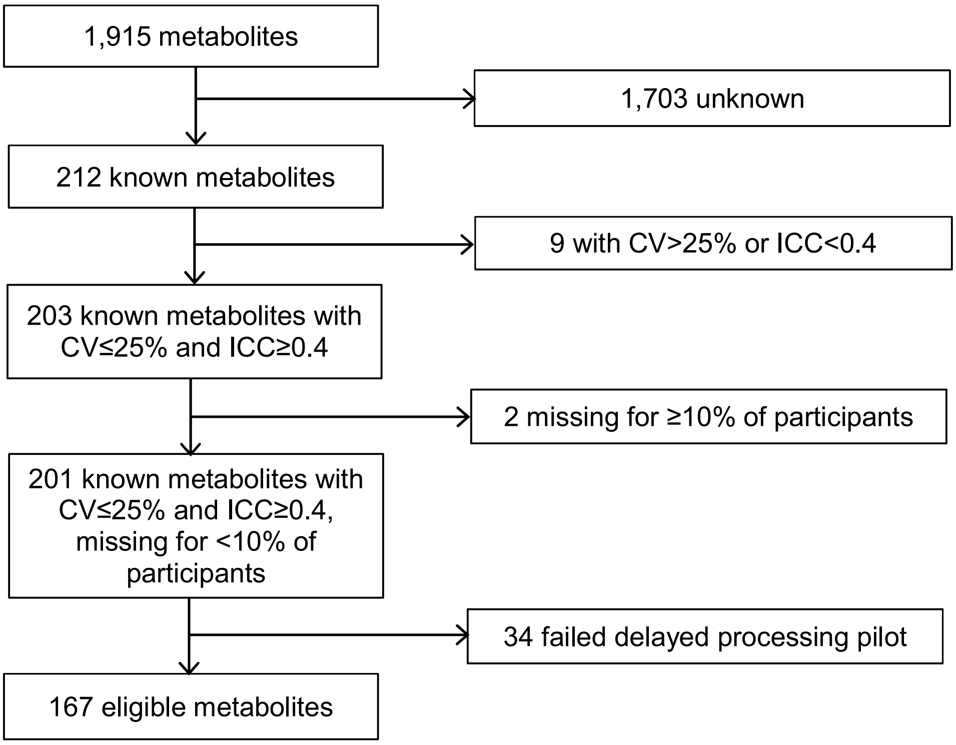

HILIC-positive method (amino acids, dipeptides, and other cationic metabolites)

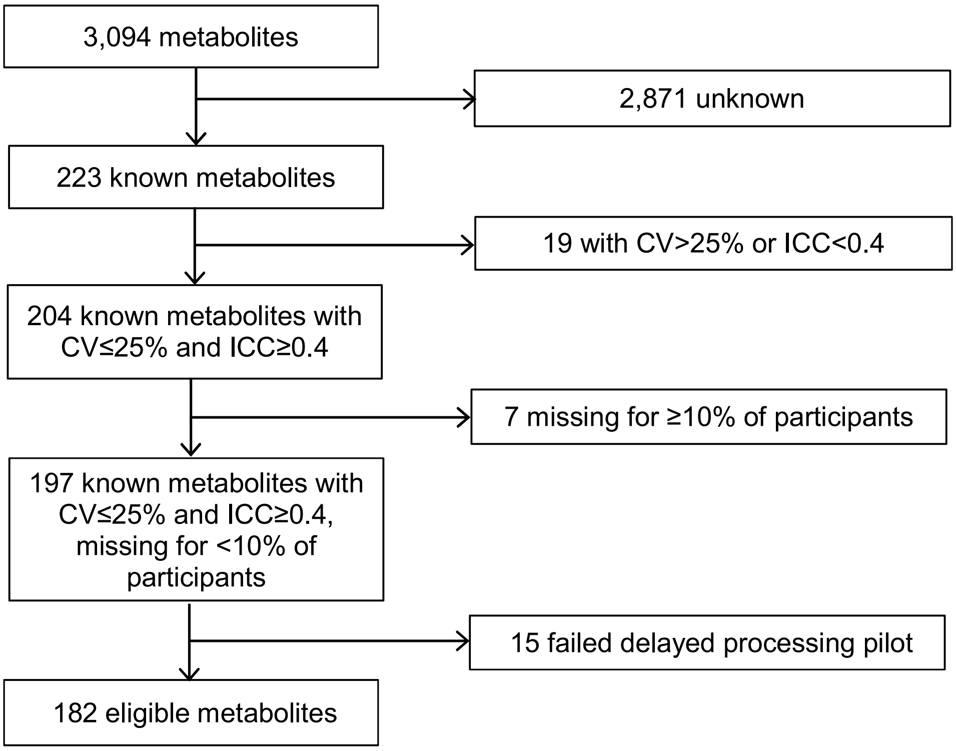

PCA nested case-control study

C8-positive method (polar and non-polar lipids)

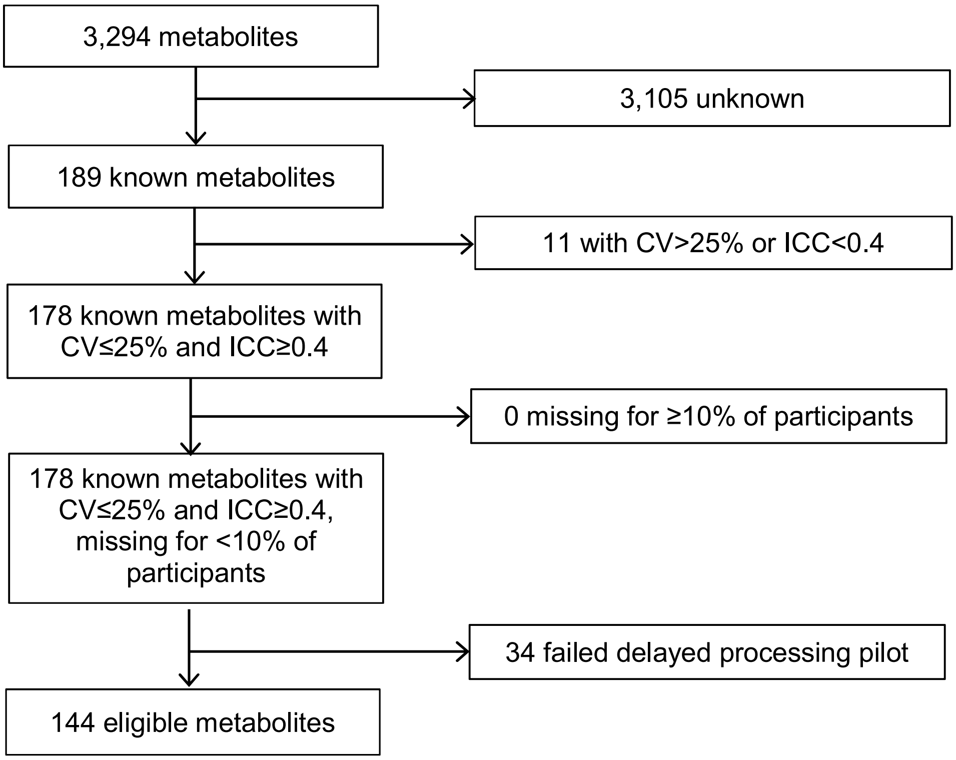

HILIC-positive method (amino acids, dipeptides, and other cationic metabolites)

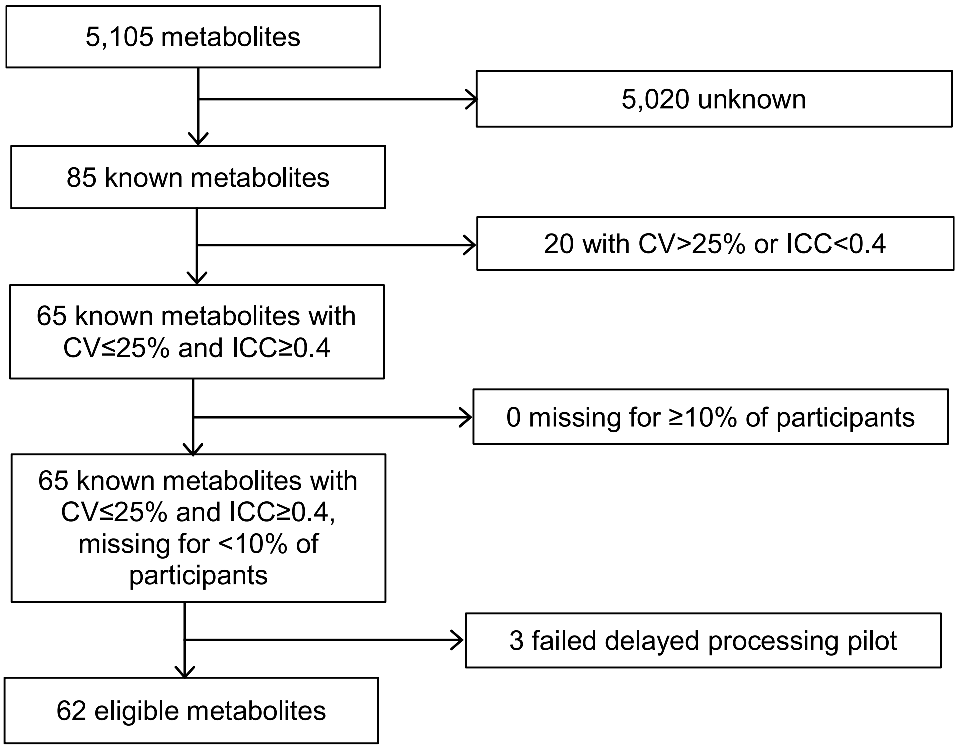

Figure S3 Overlap in identified metabolites across adiposity measures (from Main Table 2)

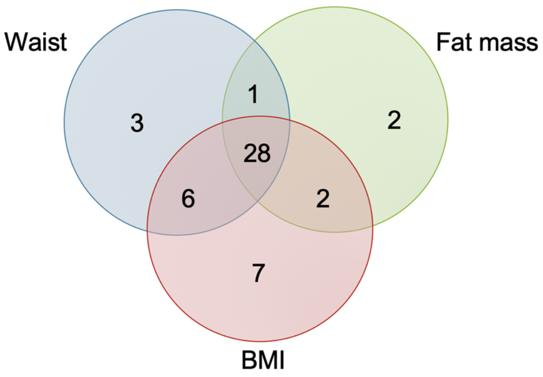

Associated with	Metabolite name		
All three measures (n=28)	HMDB10386	HMDB07098	HMDB05367
	HMDB10397	HMDB07103	HMDB07219
	HMDB00123	HMDB05384	HMDB05376
	HMDB11503	HMDB05433	HMDB00641
	HMDB02815	HMDB05377	HMDB05432
	HMDB10404	HMDB07132	HMDB10412
	HMDB07102	HMDB07218	HMDB05403
	HMDB05369	HMDB07216	HMDB05359
	HMDB07099	HMDB10375	HMDB10419
	HMDB05360		
BMI only (n=7)	HMDB11507	HMDB00688	HMDB02013
	HMDB11130	HMDB07199	HMDB11210
	HMDB11526		
Waist circumference only (n=3)	HMDB05435	HMDB05405	HMDB13326
Fat mass only (n=2)	HMDB06733	HMDB00222	
BMI and waist circumference only (n=6)	HMDB08047	HMDB00705	HMDB07248
	HMDB08057	HMDB05363	HMDB06347
BMI and fat mass only (n=2)	HMDB11506	HMDB08511	
Waist circumference and fat mass only (n=1)	HMDB03331		

Table S1 Metabolites associated with adiposity measures (FDR *p*-value <0.05 and |r| ≥0.15) among 217 controls from nested case-control studies with data on all three adiposity measures, Health Professionals Follow-up Study, 1993-1996.

		Body mass index		Waist circumference		Derived fat mass	
**HMDB ID[a]**	**Metabolite name**	**Pearson correlation coefficient[b]**	**FDR *p*-value**	**Pearson correlation coefficient[b]**	**FDR *p*-value**	**Pearson correlation coefficient[b]**	**FDR *p*-value**
**Amino acids**							
HMDB00123	glycine	-0.30	<0.001	-0.21	0.03	-0.25	0.01
HMDB00641	glutamine	-0.20	0.02	-0.18	0.05	-0.18	0.04
**Carnitines**							
HMDB00688	C5 carnitine	0.18	0.04	--	--	--	--
HMDB06347	C26 carnitine	0.18	0.04	--	--	--	--
HMDB00705	C6 carnitine	--	--	0.19	0.05	--	--
HMDB00222	C16 carnitine	--	--	--	--	0.18	0.04
**Lipids**							
CE							
HMDB10375	C22:5 CE	-0.20	0.02	-0.22	0.03	-0.24	0.01
HMDB06733	C22:6 CE	--	--	--	--	-0.19	0.04
DAG							
Saturated							
HMDB07098	C32:0 DAG	0.23	0.01	0.20	0.03	0.22	0.01
Unsaturated							
HMDB07102	C34:1 DAG	0.24	0.01	0.22	0.03	0.24	0.01
HMDB07099	C32:1 DAG	0.23	0.01	0.22	0.03	0.24	0.01
HMDB07103	C34:2 DAG	0.22	0.01	0.22	0.03	0.23	0.01
HMDB07216	C36:1 DAG	0.21	0.02	0.21	0.03	0.22	0.01
HMDB07218	C36:2 DAG	0.21	0.02	0.20	0.03	0.21	0.02
HMDB07132	C34:3 DAG	0.21	0.02	0.22	0.03	0.23	0.01
HMDB07219	C36:3 DAG	0.19	0.03	0.19	0.04	0.20	0.03

HMDB07248	C36:4 DAG	--	--	--	--	0.18	0.05
LPC							
HMDB10386	C18:2 LPC	-0.36	<0.0001	-0.24	0.03	-0.30	<0.001
HMDB10397	C20:5 LPC	-0.35	<0.0001	-0.24	0.03	-0.29	<0.001
HMDB02815	C18:1 LPC	-0.31	<0.001	-0.23	0.03	-0.27	<0.01
HMDB10404	C22:6 LPC	-0.27	<0.01	-0.21	0.03	-0.26	0.01
LPE							
HMDB11503	C16:0 LPE	-0.29	<0.001	-0.20	0.03	-0.23	0.01
HMDB11507	C18:2 LPE	-0.29	<0.001	--	--	-0.18	0.04
HMDB11506	C18:1 LPE	-0.24	0.01	--	--	-0.19	0.03
HMDB11130	C18:0 LPE	-0.21	0.02	--	--	--	--
HMDB11526	C22:6 LPE	-0.20	0.02	--	--	--	--
PC							
HMDB08047	C38:3 PC	0.21	0.02	--	--	--	--
HMDB08057	C40:6 PC	0.19	0.03	--	--	--	--
HMDB08511	C40:10 PC	--	--	--	--	-0.18	0.04
TAG							
HMDB05369	C52:2 TAG	0.24	0.01	0.21	0.03	0.22	0.01
HMDB05360	C50:1 TAG	0.23	0.01	0.19	0.04	0.22	0.01
HMDB05384	C52:3 TAG	0.22	0.01	0.20	0.03	0.21	0.02
HMDB05377	C50:2 TAG	0.22	0.01	0.19	0.04	0.21	0.02
HMDB05433	C50:3 TAG	0.21	0.02	0.20	0.03	0.22	0.01
HMDB05367	C52:1 TAG	0.21	0.02	0.19	0.04	0.21	0.02
HMDB05376	C48:2 TAG	0.19	0.03	--	--	0.20	0.02
HMDB05403	C54:2 TAG	0.18	0.04	0.19	0.05	0.19	0.03
HMDB10412	C46:1 TAG	0.18	0.05	--	--	0.19	0.04
HMDB05432	C48:3 TAG	--	--	--	--	0.19	0.03
HMDB10419	C46:2 TAG	--	--	--	--	0.18	0.04
HMDB05363	C52:4 TAG	--	--	--	--	0.18	0.04

**Purines, pyrimidines, and derivatives**							
HMDB03331	1-methyladenosine	--	--	--	--	0.19	0.04

Abbreviations: CE, cholesterol ester; DAG, diacylglycerol; LPC, lysophosphatidylcholine; LPE, lysophosphatidylethanolamine; PC, phosphatidylcholine; TAG, triacylglycerol.

[a] Representative HMDB IDs provided for PC, DAG, and TAG lipids.

[b] Estimates from partial Pearson correlation, adjusted for age (continuous) and smoking status (ever/never). 33 metabolites associated with BMI, 24 with waist circumference, 35 with derived fat mass.

Table S2 Metabolites associated with adiposity measures (FDR *p*-value <0.05 and |r| ≥0.15) among controls (n=118-126)[a] from nested case-control studies with fasting (≥8 hours) blood samples, Health Professionals Follow-up Study, 1993-1996.

		Body mass index		Waist circumference		Derived fat mass	
HMDB ID[b]	Metabolite name	Pearson correlation coefficient[c]	FDR *p*-value	Pearson correlation coefficient[c]	FDR *p*-value	Pearson correlation coefficient[c]	FDR *p*-value
**Amino acids**							
HMDB00123	glycine	-0.32	<0.01	--	--	-0.31	0.01
HMDB00883	valine	0.23	0.04	--	--	--	--
**Lipids**							
CE							
HMDB06733	C22:6 CE	-0.26	0.02	--	--	-0.28	0.01
HMDB10375	C22:5 CE	-0.24	0.03	-0.23	0.05	-0.24	0.04
Ceramide							
HMDB04952	C22:0 Ceramide (d18:1)	--	--	0.23	0.05	--	--
HMDB04953	C24:1 Ceramide (d18:1)	--	--	0.24	0.03	--	--
DAG							
Saturated							
HMDB07098	C32:0 DAG	0.36	<0.01	0.34	<0.01	0.35	<0.01
Unsaturated							
HMDB07102	C34:1 DAG	0.35	<0.01	0.35	<0.01	0.35	<0.01
HMDB07099	C32:1 DAG	0.33	<0.01	0.34	<0.01	0.34	<0.01
HMDB07216	C36:1 DAG	0.32	<0.01	0.32	<0.01	0.31	0.01
HMDB07218	C36:2 DAG	0.31	0.01	0.33	<0.01	0.31	0.01
HMDB07103	C34:2 DAG	0.31	0.01	0.33	<0.01	0.31	0.01
HMDB07132	C34:3 DAG	0.25	0.03	0.27	0.02	0.25	0.03
HMDB07219	C36:3 DAG	0.25	0.03	0.26	0.02	--	--
LPC							
HMDB10386	C18:2 LPC	-0.34	<0.01	-0.27	0.02	-0.37	<0.01

HMDB10397	C20:5 LPC	-0.33	<0.01	-0.26	0.02	-0.35	<0.01
HMDB10404	C22:6 LPC	-0.32	<0.01	-0.23	0.05	-0.37	<0.01
HMDB02815	C18:1 LPC	-0.28	0.01	--	--	-0.31	0.01
HMDB10395	C20:4 LPC	--	--	--	--	-0.25	0.03
LPE							
HMDB11503	C16:0 LPE	-0.26	0.02	--	--	-0.26	0.02
HMDB11526	C22:6 LPE	-0.26	0.02	--	--	-0.25	0.04
HMDB11507	C18:2 LPE	-0.26	0.02	--	--	--	--
PC							
HMDB08047	C38:3 PC	0.27	0.01	0.28	0.01	0.25	0.03
HMDB08057	C40:6 PC	0.25	0.03	0.27	0.02	--	--
TAG							
Saturated							
HMDB05365	C52:0 TAG	0.30	0.01	0.29	0.01	0.27	0.02
HMDB42063	C44:0 TAG	0.30	0.01	0.28	0.01	0.30	0.01
HMDB05356	C48:0 TAG	0.29	0.01	0.25	0.02	0.27	0.02
HMDB05357	C50:0 TAG	0.28	0.01	0.25	0.02	0.26	0.03
HMDB10411	C46:0 TAG	0.27	0.01	0.24	0.03	0.27	0.02
Unsaturated							
HMDB05360	C50:1 TAG	0.36	<0.01	0.35	<0.01	0.36	<0.01
HMDB05377	C50:2 TAG	0.35	<0.01	0.35	<0.01	0.35	<0.01
HMDB05369	C52:2 TAG	0.34	<0.01	0.34	<0.01	0.34	<0.01
HMDB05367	C52:1 TAG	0.33	<0.01	0.32	<0.01	0.32	0.01
HMDB05433	C50:3 TAG	0.33	<0.01	0.34	<0.01	0.33	<0.01
HMDB05376	C48:2 TAG	0.31	0.01	0.32	<0.01	0.32	0.01
HMDB05359	C48:1 TAG	0.31	0.01	0.30	0.01	0.32	0.01
HMDB10412	C46:1 TAG	0.30	0.01	0.29	0.01	0.31	0.01
HMDB05395	C54:1 TAG	0.30	0.01	0.32	<0.01	0.29	0.01
HMDB05403	C54:2 TAG	0.30	0.01	0.33	<0.01	0.30	0.01

HMDB05384	C52:3 TAG	0.28	0.01	0.29	0.01	0.27	0.02
HMDB10419	C46:2 TAG	0.28	0.01	0.29	0.01	0.29	0.01
HMDB05432	C48:3 TAG	0.27	0.01	0.30	0.01	0.29	0.01
HMDB05404	C56:2 TAG	0.24	0.04	0.26	0.02	--	--
HMDB05435	C50:4 TAG	--	--	0.26	0.02	--	--
HMDB05410	C56:3 TAG	--	--	0.24	0.03	--	--
HMDB05405	C54:3 TAG	--	--	0.26	0.02	--	--
**Nitrogenous organic acid**							
HMDB00064	creatine	0.24	0.04	--	--	--	--

Abbreviations: CE, cholesterol ester; DAG, diacylglycerol; LPC, lysophosphatidylcholine; LPE, lysophosphatidylethanolamine; PC, phosphatidylcholine; TAG, triacylglycerol.

[a] The number of men contributing to each analysis was 125 for BMI, 126 for waist circumference, 118 for derived fat mass.

[b] Representative HMDB IDs provided for PC, DAG, and TAG lipids.

[c] Estimates from partial Pearson correlation, adjusted for age (continuous) and smoking status (ever/never). 41 metabolites associated with BMI, 38 with waist circumference, 36 with derived fat mass.

File S2. Lasso models used for predicting adiposity using associated metabolites

Model 1 BMI model

Dependent variable: BMI (kg/m^2)

```
(Intercept)    25.531249633
Z_LN_HMDB02815  .
Z_LN_HMDB05359  .
Z_LN_HMDB05360  .
Z_LN_HMDB05363  .
Z_LN_HMDB05367  0.035434842
Z_LN_HMDB05369  .
Z_LN_HMDB05376  .
Z_LN_HMDB05377  .
Z_LN_HMDB05384  .
Z_LN_HMDB05403  .
Z_LN_HMDB05432  .
Z_LN_HMDB05433  .
Z_LN_HMDB07098  0.327006229
Z_LN_HMDB07099  .
Z_LN_HMDB07102  .
Z_LN_HMDB07103  .
Z_LN_HMDB07132  .
Z_LN_HMDB07199  0.002574509
Z_LN_HMDB07216  .
Z_LN_HMDB07218  .
Z_LN_HMDB07219  .
Z_LN_HMDB07248  0.149999338
Z_LN_HMDB08047  0.286568241
Z_LN_HMDB08057  .
Z_LN_HMDB08511 -0.097942617
Z_LN_HMDB10375  .
Z_LN_HMDB10386 -0.144544559
Z_LN_HMDB10397 -0.082997714
Z_LN_HMDB10404  .
Z_LN_HMDB10412  0.099054476
Z_LN_HMDB10419  .
Z_LN_HMDB11130  .
Z_LN_HMDB11210  .
Z_LN_HMDB11503 -0.670392913
Z_LN_HMDB11506  .
Z_LN_HMDB11507 -0.183100200
Z_LN_HMDB11526  .
Z_LN_HMDB00123 -0.242880442
Z_LN_HMDB00641 -0.228753242
Z_LN_HMDB00688  .
Z_LN_HMDB00705  .
Z_LN_HMDB02013  0.174925769
Z_LN_HMDB06347  0.272082992
```

Model 2 Waist circumference model

Dependent variable: Waist circumference (cm)

```
(Intercept)    95.2876910
Z_LN_HMDB02815 -0.2607707
Z_LN_HMDB05359  .
```

Z_LN_HMDB05360	.
Z_LN_HMDB05363	.
Z_LN_HMDB05367	.
Z_LN_HMDB05369	.
Z_LN_HMDB05376	.
Z_LN_HMDB05377	.
Z_LN_HMDB05384	.
Z_LN_HMDB05403	0.2957983
Z_LN_HMDB05405	.
Z_LN_HMDB05432	.
Z_LN_HMDB05433	.
Z_LN_HMDB05435	.
Z_LN_HMDB07098	.
Z_LN_HMDB07099	0.2622919
Z_LN_HMDB07102	0.3253620
Z_LN_HMDB07103	.
Z_LN_HMDB07132	.
Z_LN_HMDB07216	0.3920670
Z_LN_HMDB07218	.
Z_LN_HMDB07219	.
Z_LN_HMDB07248	.
Z_LN_HMDB08047	0.4903875
Z_LN_HMDB08057	.
Z_LN_HMDB10375	-0.2535561
Z_LN_HMDB10386	-0.5293353
Z_LN_HMDB10397	.
Z_LN_HMDB10404	-0.3723265
Z_LN_HMDB10412	.
Z_LN_HMDB10419	.
Z_LN_HMDB11503	-0.7386762
Z_LN_HMDB00123	.
Z_LN_HMDB00641	-1.0275656
Z_LN_HMDB00705	0.1027872
Z_LN_HMDB03331	0.8122641
Z_LN_HMDB06347	1.0358597
Z_LN_HMDB13326	0.5154372

Model 3 Fat mass model

Dependent variable: Derived fat mass (kg)

```
(Intercept)    21.7917898823
Z_LN_HMDB02815 -0.4020370190
Z_LN_HMDB05359 -1.5255249821
Z_LN_HMDB05360  .
Z_LN_HMDB05367  1.1497788112
Z_LN_HMDB05369  .
Z_LN_HMDB05376  .
Z_LN_HMDB05377  .
Z_LN_HMDB05384 -0.7831811168
Z_LN_HMDB05403  .
Z_LN_HMDB05432  1.3358576755
Z_LN_HMDB05433  .
Z_LN_HMDB06733  1.0052774695
Z_LN_HMDB07098  0.0157630573
Z_LN_HMDB07099  .
Z_LN_HMDB07102  0.5981972979
Z_LN_HMDB07103 -0.0919940811
Z_LN_HMDB07132  0.0001796992
Z_LN_HMDB07216  .
Z_LN_HMDB07218  .
Z_LN_HMDB07219  0.3776334578
Z_LN_HMDB08511 -0.3002406728
Z_LN_HMDB10375 -0.5037533119
Z_LN_HMDB10386  .
Z_LN_HMDB10397  0.0347104906
Z_LN_HMDB10404 -1.0242727239
Z_LN_HMDB10412  0.4452927910
Z_LN_HMDB10419  .
Z_LN_HMDB11503 -0.7353253207
Z_LN_HMDB11506  .
Z_LN_HMDB00123 -0.4303850733
Z_LN_HMDB00222  1.1136072086
Z_LN_HMDB00641 -0.5580088922
Z_LN_HMDB03331  0.2424098130
```
